# The Evolution of Vision Therapy Software and Its Impact on Vision Care - A Comprehensive Major Review

**DOI:** 10.22336/rjo.2025.78

**Published:** 2025

**Authors:** Inbarasi Nagendran, Lipika Roy, Pavitra Ravikumar, Prasanth Gireesh, Kirandeep Kaur, Bharat Gurnani

**Affiliations:** 1 Department of Vision Therapy, Hexa Vision Enhancement Centre, Chennai, Tamil Nadu, India; 2 Department of Pediatric Ophthalmology, Hexa Vision Enhancement Centre, Chennai, Tamil Nadu, India; 3 Department of Pediatric Ophthalmology, Centre for Vision and Eye Surgery, Chennai, Tamil Nadu, India; 4 Department of Cataract, Hexa Vision Enhancement Centre, Chennai, Tamil Nadu, India; 5 Department of Cataract, Pediatric Ophthalmology and Strabismus, Gomabai Netralaya and Research Centre, Neemuch, Madhya Pradesh, India; 6 Department of Cataract, Cornea and Refractive Surgery, Gomabai Netralaya and Research Centre, Neemuch, Madhya Pradesh, India

**Keywords:** vision therapy software, amblyopia, strabismus, binocular vision disorders, AI-driven vision rehabilitation, VR-based vision therapy, digital eye care, AI = Artificial Intelligence, AOA = American Optometric Association, AR = Augmented Reality, CI = Convergence Insufficiency, CPT = Computerized Perceptual Therapy, GDPR = General Data Protection Regulation, GRADE = Grading of Recommendations, Assessment, Development, and Evaluations, HIPAA = Health Insurance Portability and Accountability Act, PSVPP = Primary Sports Vision Performance Profile, PTS = Perceptual Therapy System, SVI = Sanet Vision Integrator, VR = Virtual Reality, VTS = Vision Therapy Software

## Abstract

The integration of vision therapy software (VTS) has brought about a paradigm shift in the treatment of various visual disorders, enhancing patient engagement, therapy effectiveness, and accessibility. The advent of VTS has revolutionized the management of binocular vision disorders, amblyopia, convergence insufficiency, and post-traumatic visual dysfunctions, providing a technologically advanced, accessible, and engaging alternative to traditional in-office therapy. VTS leverages artificial intelligence (AI), virtual reality (VR), augmented reality (AR), and wearable technologies to create interactive, adaptive, and personalized training environments that enhance neuroplasticity and visual rehabilitation. This review explores the evolution, mechanisms, and clinical applications of VTS, emphasizing its role in improving visual acuity, binocular coordination, vergence, and accommodative functions. Evidence from meta-analyses, clinical trials, and case studies supports its effectiveness, with notable benefits in patient adherence, treatment flexibility, and cost reduction. Additionally, telemedicine integration and remote monitoring have expanded access to vision therapy, particularly in underserved and remote populations. Despite its advantages, challenges remain, including the need for professional supervision, variability in patient response, lack of standardization, and data privacy concerns. Furthermore, long-term comparative studies are necessary to validate its efficacy against traditional vision therapy methods. Emerging AI-driven personalization, immersive VR/AR environments, and real-time biofeedback mechanisms are expected to enhance VTS, making it a crucial component of modern vision rehabilitation. As digital therapeutics continue to evolve, a hybrid model combining VTS with clinician-guided therapy appears to be the most effective approach. This paradigm shift underscores VTS’s future potential to redefine vision care and bridge the gap between clinical expertise and innovative digital health solutions.

## Introduction

Vision therapy (VT) is a structured, evidence-based approach to enhancing visual function through specialized exercises designed to improve binocular vision, eye tracking, focusing, and coordination [**1**]. Traditionally, vision therapy has been conducted in clinical settings under professional supervision, using manual techniques, optical devices, and physical exercises [**2**]. However, with advancements in digital health technology, vision therapy software (VTS) has emerged as a game-changing innovation, bringing therapy beyond clinical walls into patients’ homes. Vision therapy software is computer-based, AI-driven, and interactive, providing personalized, engaging, and adaptive programs for vision training [**3**]. These platforms utilize virtual reality (VR), augmented reality (AR), gamification, and artificial intelligence (AI) to create dynamic and immersive training environments. The goal of VTS is to stimulate neuroplasticity—the brain’s ability to reorganize and form new neural connections—thus improving visual processing, eye alignment, and coordination [**4**]. Unlike conventional vision therapy, which often requires frequent in-office visits, software-based therapy enables remote, home-based training, making vision care more accessible, cost-effective, and engaging [**5**]. Vision therapy software has gained momentum due to the increased prevalence of binocular vision disorders and digital eye strain, particularly amid rising screen exposure and prolonged near-work activities [**6**]. Studies indicate that amblyopia (lazy eye) affects approximately 3% of children worldwide, while convergence insufficiency (CI) is prevalent in up to 13% of the population. Moreover, post-concussion vision problems, digital eye strain, and accommodative dysfunctions are becoming increasingly common [**7**]. Traditional vision therapy methods, though effective, face barriers such as cost, patient compliance, and geographical limitations. Digital therapy solutions address these challenges by providing on-demand, structured, and data-driven therapy, improving both adherence and clinical outcomes [**8**]. Modern VTS platforms leverage AI-based algorithms to assess patient progress and adapt therapy in real time, ensuring that exercises remain challenging yet achievable. These programs often include: Binocular Vision Training – Exercises designed to improve eye teaming, fusion, and depth perception, frequently using stereoscopic or dichoptic stimuli to strengthen binocular function [**9**]. Saccadic & Pursuit Training – Programs targeting eye tracking and smooth pursuit movements, essential for reading fluency and sports vision. Vergence & Accommodation Therapy – Tools to improve the ability to shift focus between near and far objects, benefiting patients with convergence insufficiency and accommodative dysfunctions [**10**]. AI-Powered Personalization – Intelligent software that adjusts difficulty levels based on user performance, ensuring a tailored therapeutic experience. Wearable Integration – Use of VR headsets, smart glasses, and eye-tracking devices to provide real-time feedback and enhance immersion [**11**]. Several well-known vision therapy software platforms have demonstrated efficacy in treating a wide range of conditions, including amblyopia (lazy eye). Programs like RevitalVision and Luminopia employ dichoptic training to encourage both eyes to work together, reducing suppression of the weaker eye [**12**]. Strabismus & Binocular Vision Disorders: VR-based therapy, such as Vivid Vision, provides interactive depth perception training for patients with intermittent exotropia and convergence insufficiency. Convergence Insufficiency & Digital Eye Strain: AmblyoPlay and Optics Trainer offer targeted exercises for eye coordination and near-point focusing, essential for screen users and students. Post-Traumatic Vision Disorders (TBI & Concussion): NeuroTracker and RightEye provide neuro-optometric rehabilitation to improve visual tracking, cognitive function, and reaction time [**13**]. Myopia Control & Accommodative Disorders: Emerging research suggests that vision therapy software can help reduce accommodative stress, potentially aiding in controlling myopia progression [**14**].

### The key advantages of vision therapy software over traditional methods

Accessibility & Convenience: Enables home-based therapy, reducing the need for frequent clinic visits. Personalization: AI-driven adaptive difficulty levels cater to individual needs, enhancing efficacy [**15**]. Engagement & Compliance: Gamified, interactive exercises increase patient motivation, particularly among children. Cost-Effectiveness: Eliminates travel expenses and time constraints, making therapy more affordable. Remote Monitoring & Data-Driven Insights: Clinicians can track patient progress remotely, allowing real-time therapy adjustments [**16**]. There are a few challenges and limitations to the effectiveness of Vision Therapy Software compared to Traditional In-Office Therapy. In some cases, such as severe strabismus or post-surgical rehabilitation, in-person therapy may be required for optimal results. Need for Professional Supervision: Patients may struggle to execute techniques, necessitating clinical guidance. Variability in Patient Response: Factors such as age, compliance, and cognitive engagement influence therapy effectiveness [**1**]. Data Privacy & Ethical Concerns: The collection of sensitive patient data raises concerns about security, AI bias, and regulatory compliance (HIPAA, GDPR). The future of vision therapy software is driven by AI, VR, AR, and wearable technology: AI & Machine Learning: Intelligent algorithms will optimize therapy customization and predict treatment outcomes [**17**]. Virtual Reality (VR) & Augmented Reality (AR) - Immersive environments will enhance binocular vision training with real-world simulation. Wearable Tech & Eye-Tracking: Real-time biofeedback through smart glasses and mobile devices will improve therapy precision. Telemedicine & Remote Monitoring: Cloud-based platforms will enable seamless integration with optometric and ophthalmologic care. Vision therapy software represents a paradigm shift in vision care, bridging the gap between clinical vision therapy and digital health innovation. By making therapy accessible, engaging, and data-driven, VTS is reshaping the way binocular vision disorders, amblyopia, and accommodative dysfunctions are managed. While challenges remain, the integration of AI, VR, and telehealth will continue to enhance the effectiveness and reach of digital vision rehabilitation, paving the way for personalized, scalable, and innovative eye care [**18**].

### Literature search strategy

A systematic, structured literature search strategy is essential for compiling a high-quality, evidence-based review of vision therapy software (VTS). The first step is to define a clear research question, such as “What is the efficacy, clinical applications, and future advancements of vision therapy software in managing binocular vision disorders, amblyopia, and related conditions?” Next, a comprehensive search strategy should incorporate a combination of keywords and Boolean operators, including terms such as “vision therapy software”, “digital vision therapy”, “computer-based vision therapy”, and “amblyopia”, or “binocular vision disorders” or “convergence insufficiency”, to ensure a broad yet focused search. Relevant academic databases, such as PubMed, Scopus, Web of Science, IEEE Xplore, Cochrane Library, and Google Scholar, should be systematically searched, applying filters for study type, date range (preferably the last 5–10 years), and language (English-only for accessibility and consistency). Inclusion criteria should focus on meta-analyses, clinical trials, comparative studies, and systematic reviews that evaluate the efficacy, patient compliance, technological integration (AI, VR, AR, wearable eye-tracking), and economic impact of VTS. Studies that do not directly involve digital or software-based vision therapy, lack peer review, or have insufficient sample sizes should be excluded to maintain research validity. Citation tools such as Zotero, Mendeley, or EndNote should be used to efficiently manage and organize references. Furthermore, the PRISMA (Preferred Reporting Items for Systematic Reviews and Meta-Analyses) framework should guide the screening, selection, and reporting process to enhance transparency and reproducibility. After data extraction, a critical appraisal using the GRADE (Grading of Recommendations, Assessment, Development, and Evaluations) system will help assess the strength of the evidence. The findings should be synthesized into thematic categories such as clinical effectiveness, patient adherence, future trends in AI-driven therapy, and telemedicine integration, followed by an in-depth discussion on challenges, gaps, and future research directions. By following this rigorous literature search strategy, the review article will provide a comprehensive, well-supported analysis of vision therapy software, offering valuable insights into digital vision rehabilitation.

### Evolution of vision therapy software

Vision therapy software has emerged from the need for more scalable, efficient, and engaging methods to treat visual disorders. Early software programs were simple computer games designed to encourage visual tracking and eye-hand coordination [**19**]. Over time, advancements in artificial intelligence, virtual reality (VR), and augmented reality technologies have led to the development of sophisticated platforms that simulate real-world tasks, gamify exercises, and track patient progress with high precision. The literature review on vision therapy software highlights diverse advancements and their implications across studies [**20**]. Overall, the studies collectively underscore the potential of vision therapy software while identifying limitations and recommending avenues for further research to optimize its effectiveness across diverse populations and conditions [**21**].

### Historical perspective: conventional vision therapy

Vision therapy has a long history, rooted in orthoptics, which emerged in the early 20th century as a method for treating strabismus and binocular vision anomalies. Initially, eye exercises and patching techniques were used to strengthen eye muscles and improve visual coordination [**22**]. In the mid-1900s, the field expanded beyond orthoptics, with optometrists recognizing that vision therapy could be applied to a broader range of visual dysfunctions, including convergence insufficiency, accommodative disorders, and learning-related vision problems. The introduction of lens-based therapy, prisms, and occlusion therapy helped refine treatment strategies [**23**]. By the 1970s and 1980s, behavioral optometry gained prominence, emphasizing the neurological component of vision. This shift moved vision therapy beyond simply training eye muscles to addressing visual processing and integration with cognitive and motor functions [**24**]. **Table 1** depicts the key milestones in Vision Therapy Development.

**Table 1:** Key milestones in vision therapy development

S. No	Year	Development Stage	Key Developments
1	Early 1900s	Foundation of Orthoptics	Pioneered as a clinical discipline focusing on eye muscle training and binocular vision disorders. The first orthoptic clinics were established using basic exercises to improve eye alignment and coordination.
2	Mid-20th Century	Expansion Beyond Strabismus Treatment	Introduction of vergence therapy for binocular dysfunction. Emergence of optometric vision therapy to treat accommodation, convergence, and perceptual issues.
3	1970s–1980s	Behavioral Optometry and Neuroplasticity	Research highlighted the role of vision in learning disabilities and sports performance. Development of perceptual-motor therapy, integrating vision training with sensory-motor functions.
4	1990s	Evidence-Based Approaches and Professional Recognition	Studies have confirmed the efficacy of vision therapy in conditions such as convergence insufficiency (CITT Study). Organizations such as the American Optometric Association (AOA) endorsed structured therapy programs.
5	2000s	Digital Transformation Begins	Computer-based vision therapy programs were introduced for binocular vision training and neuro-optometric rehabilitation. Use of video games and interactive stimuli to enhance compliance and engagement.
6	2010s–Present	Emergence of Vision Therapy Software & AI Integration	Development of cloud-based and AI-driven therapy programs. Introduction of virtual reality (VR) and augmented reality (AR) for immersive vision rehabilitation. Increased accessibility via remote therapy platforms, allowing home-based treatments.

### Integration of technology into eye care

The adoption of digital interfaces has transformed traditional exercises into interactive programs that adapt difficulty levels to patient progress. Software such as RevitalVision, HTS Vision Therapy, and Optics Trainer provide structured, data-driven therapy [**25**]. AI-powered platforms analyze patient responses in real time and adapt therapy plans accordingly. Machine learning models help predict treatment outcomes and optimize therapy sessions [**26**]. Virtual Reality (VR) headsets create immersive environments for amblyopia, binocular dysfunction, and convergence training. Augmented Reality (AR) overlays provide real-time guidance for ocular exercises, making therapy more engaging [**27**]. Cloud-based software enables patients to complete therapy at home, reducing the need for clinic visits. Eye-tracking and digital feedback tools help clinicians monitor progress remotely. Devices like smart glasses and eye-tracking sensors provide real-time biofeedback, enhancing therapy effectiveness. Emerging tools allow continuous monitoring of eye movements and accommodation dynamics [**28**]. The evolution of vision therapy from manual exercises to AI-driven, VR-integrated platforms marks a paradigm shift in vision care. With technology enhancing accessibility, personalization, and engagement, modern vision therapy is poised to revolutionize the management of visual dysfunctions, improving patient outcomes more efficiently and at scale [**29**].

### Vision therapy software

Vision therapy software represents a cutting-edge intersection of technology and vision rehabilitation, offering interactive digital solutions to improve visual skills and address a range of binocular vision disorders [**30**]. Below is an overview that defines these tools, outlines their core features, discusses their types, and highlights popular software applications currently in use. Vision therapy software refers to computer-based programs and applications that deliver structured, interactive exercises to enhance visual skills, such as eye teaming, tracking, focusing, and depth perception [**31**]. Vision therapists, optometrists, and educators use these digital platforms to design personalized treatment plans for individuals with conditions like strabismus, amblyopia, convergence insufficiency, and other binocular vision anomalies [**32**].

#### Core features

**Customization and Adaptability are the primary core features**. Programs typically allow practitioners to tailor exercises to each patient’s specific needs and progress levels. Adjustments can be made to difficulty, speed, contrast, and other parameters [**33**]. Many vision therapy programs incorporate game-like elements, interactive and engaging exercises, and dynamic visual stimuli, which not only motivate patients but also provide real-time feedback. Comprehensive data tracking of patient performance over time enables therapists to monitor improvements, adjust treatment protocols, and quantify outcomes [**34**]. User-friendly interfaces make it easier for patients to use the software in both clinical settings and at home, thereby enhancing treatment adherence and consistency. Some platforms support hardware such as virtual reality headsets, tablets, and specialized vision-training devices to provide a more immersive experience [**35**].

### Types of vision therapy software

Vision therapy software is generally categorized based on the specific visual skills it aims to enhance. Here are some common types:

### Binocular vision training software

Focuses on improving the coordination between both eyes. These programs help in enhancing convergence (the inward movement of the eyes when focusing on a near object) and divergence, which are critical for depth perception and overall visual comfort.

#### Dichoptic presentation

Exercises may present different images to each eye, forcing the brain to integrate disparate visual information.

#### Alignment and fusion tasks

Activities are designed to improve the ability to align the visual axes and fuse images from both eyes, which is essential for binocular vision [**36**].

### Saccadic and pursuit training programs

Designed to refine eye movement control. Saccadic training targets quick, simultaneous movements of both eyes between points, while pursuit training focuses on the smooth tracking of moving objects.

#### Rapid shift exercises

Tasks require users to quickly switch focus from one target to another, thereby training saccadic eye movements.

#### Smooth tracking drills

Programs often include moving targets that require continuous tracking, enhancing the ability to maintain focus on moving objects.

#### Attention and coordination

Many exercises are structured to improve overall visual attention, processing speed, and coordination between visual and motor functions [**37**].

### Vergence and accommodation training tools

These tools are specifically aimed at improving the ability to adjust the eyes’ focus for different distances (accommodation) and to maintain proper alignment (vergence).

#### Dynamic focus tasks

Exercises may involve shifting focus between near and far targets, simulating real-world demands on the visual system.

#### Depth perception exercises

Programs are designed to enhance the brain’s ability to judge distance, an essential skill for activities such as reading or driving.

#### Adaptive difficulty levels

Many tools automatically adjust the level of challenge based on the user’s performance, ensuring a consistent progression in visual training [**38**].

### Software platforms in the current application

Several vision therapy software platforms have gained recognition for their efficacy and innovation. Some of the most popular include:

#### Vivid vision

Utilizes virtual reality to immerse patients in a 3D environment, offering a wide array of binocular vision exercises designed to treat conditions such as amblyopia and strabismus. Its interactive games and real-time feedback are central to its appeal [**39**].

#### Optics trainer

Focuses on a range of visual skills, including depth perception and eye coordination. Known for its versatility, this software is used in both clinical and home settings, providing customizable programs that address specific vision issues.

#### AmblyoPlay

Designed specifically to support the treatment of amblyopia (lazy eye), AmblyoPlay employs engaging, game-like exercises to encourage the use of the weaker eye, gradually strengthening its performance and improving overall binocular function.

#### RevitalVision

Emphasizes a comprehensive approach to visual rehabilitation, offering modules that address eye tracking, focusing, and depth perception. It is particularly valued for its evidence-based exercises and adaptability to individual therapy needs [**40**].

Each of these platforms brings unique strengths to the field of vision therapy, reflecting ongoing advances in both technology and our understanding of visual neuroscience. Their shared goal is to provide engaging, effective, and personalized treatment options that enhance visual performance and quality of life [**41**]. Vision therapy software is transforming the landscape of vision rehabilitation by leveraging technology to deliver dynamic, personalized treatment programs. Whether through enhancing binocular coordination, refining eye movements, or improving the accommodation-vergence system, these digital tools are at the forefront of modern vision therapy, offering hope and improved outcomes for patients with a wide range of visual challenges [**42**].

### Mechanism of action: how vision therapy software works

Vision therapy software harnesses the power of modern technology and neuroscience to improve visual function. Its mechanism of action relies on several interrelated principles that work together to rewire and rehabilitate the visual system. Below is an exploration of how these software platforms operate, focusing on neuroplasticity, the use of digital stimuli with adaptive training models, the role of customization, and AI-based personalized therapy plans [**41**].

### Neuroplasticity and visual rehabilitation

**Neuroplasticity** is the brain’s ability to reorganize itself by forming new neural connections. Vision therapy software capitalizes on this principle in several ways [**43**]:

#### Stimulating neural pathways

By presenting a variety of visual tasks and challenges, the software activates and strengthens underused neural circuits related to vision. This consistent stimulation encourages the brain to adapt, forming new pathways that can compensate for deficits or inefficiencies in the visual system [**44**].

#### Rehabilitating impaired functions

Conditions like amblyopia, strabismus, or convergence insufficiency often stem from disrupted neural communication between the eyes and the brain. The targeted exercises provided by vision therapy software promote the re-establishment of normal visual processing by challenging and retraining these circuits, thus leading to functional improvements [**45**].

#### Progressive learning

The brain’s ability to adapt means that with repeated, consistent exposure to specific visual tasks, patients can gradually improve their visual performance. This concept underpins many therapy programs that introduce incremental challenges to push the boundaries of the patient’s current capabilities [**46**].

### Digital stimuli and adaptive training models

At the heart of vision therapy software is the delivery of controlled, varied digital stimuli specifically designed to target different aspects of visual function [**47**].

#### Controlled visual challenges

The software provides exercises that require users to engage in activities such as tracking moving objects, rapidly shifting focus between targets (saccadic movements), or adjusting focus between different distances (vergence and accommodation). These challenges are designed to mimic real-world visual demands [**48**].

#### Adaptive training models

Vision therapy software often uses adaptive algorithms that automatically adjust task difficulty based on the user’s performance. This ensures that the exercises remain challenging enough to stimulate improvement without overwhelming the patient [**49**]. Feedback Loops: Real-time feedback is integrated into the training sessions, helping patients understand their progress and motivating them to improve. This immediate response enables on-the-fly adjustments, ensuring each session is both effective and engaging [**50**]. Task Variability: To prevent plateauing and maintain user engagement, adaptive models introduce task variability. This variability encourages the brain to develop flexibility and resilience in its processing of visual information [**51**]. **Engaging Interfaces:** Incorporating game-like elements and immersive environments—sometimes using virtual or augmented reality—helps keep patients motivated. The engaging nature of these digital stimuli not only enhances compliance with therapy regimens but also provides a fun and interactive way to challenge the visual system [**52**].

### Customization and artificial intelligence-based personalized therapy plans

Customization is critical in vision therapy, as every patient’s visual profile and progress are unique. Modern vision therapy software often integrates **AI** to provide personalized treatment plans [**53**]. Personalized Assessment: AI algorithms can analyze a patient’s baseline performance during initial assessments, identifying specific areas of weakness and the most effective exercises to target them [**54**]. **Dynamic Adjustment:** As the patient engages with the software, the AI continuously monitors progress and adapts the therapy plan in real time. This dynamic adjustment ensures that the level of difficulty is consistently matched to the patient’s evolving abilities, leading to more efficient and effective therapy [**55**]. **Data-Driven Insights:** By aggregating data from multiple sessions, AI can identify patterns and predict which exercises yield the best outcomes for individual users. This data-driven approach enables fine-tuning of therapy protocols over time, ensuring the rehabilitation process remains optimized for each patient [**56**]. **Enhanced Patient Engagement:** Customization ensures that the exercises remain relevant and appropriately challenging, reducing frustration and improving overall engagement. Personalized therapy plans help maintain motivation and commitment to the therapy process, which is crucial for long-term success [**57**].

Vision therapy software leverages the brain’s inherent neuroplasticity through a series of targeted, adaptive, and engaging digital exercises. By delivering controlled visual stimuli, employing adaptive training models, and offering AI-driven personalized therapy plans, these platforms provide an effective means of rehabilitating and enhancing visual function [**58**]. The integration of these mechanisms not only facilitates the retraining of the visual system but also ensures that therapy is both individualized and responsive to each patient’s unique needs, ultimately leading to improved visual outcomes [**59**].

### Comparison between conventional and software-based vision therapy

A comparison of conventional and software-based vision therapy reveals clear distinctions in patient engagement, tracking, accessibility, and effectiveness. While traditional treatment requires physical objects and therapist involvement, software-based approaches employ dynamic, gamified, and data-driven tasks that increase patient compliance and engagement [**60**]. **Table 2** presents a comparison of conventional and software-based vision therapy.

**Table 2:** The comparison between conventional and software-based vision therapy

S. No	Criteria	Traditional Vision Therapy	Vision Therapy Software
1	Cost	Moderate to high due to in-office visits	The initial software cost is lower in the long term
2	Compliance	Lower, especially in children	Higher due to interactive features
3	Effectiveness in Adults	Limited, especially for amblyopia	Improved, especially with neuroplasticity-focused software
4	Duration of Effectiveness	Often requires extended therapy	Shorter, with measurable outcomes
5	Regression	Possible, especially without continued therapy	Reduced with home-based maintenance programs
6	Stereoacuity/Fusion Development	Moderate to slow	Faster with dynamic, 3D environments
7	Patient Engagement	Low to moderate	High due to gamification and real-time feedback

### Software for specific visual conditions

Vision therapy software has proven particularly effective in treating conditions such as amblyopia, strabismus, sports vision, binocular vision disorders, neurological disorders, and reading disabilities [**21**]. Platforms like Curesee, Revital Vision, and FitLight Trainer offer tailored exercises to address specific conditions and promote neuroplasticity, even in adult patients. Conventional treatments for strabismus include Brock string exercises, a synoptophore, and surgery [**61**]. These methods focus on improving binocular coordination, fusion, and ocular misalignment. While effective, they often require intense clinical supervision and are associated with inconsistent patient compliance, particularly in young children [**62**]. At the same time, software-based treatment methods offer a gamified approach that enhances patient engagement. For example, software such as Curesee, bynocs, and others provide anti-suppression, dichoptic training, perceptual learning exercises, and fusion activities designed to correct alignment through neuroplasticity [**63**]. Studies have shown that software-based methods reduce treatment duration and improve compliance by offering more interactive and less repetitive tasks [**64**]. Patching remains a cornerstone for amblyopia treatment, forcing the brain to rely on the weaker eye. However, low patient compliance, particularly due to the discomfort and social stigma associated with patching, has limited its effectiveness. Pleoptics training is another traditional method that uses specialized visual stimuli to train the amblyopic eye [**65**]. AmblyoPlay, AmbPiNet, and Revital Vision have introduced innovative ways to treat amblyopia using perceptual learning, eye-hand coordination, dichoptic training, monocular fixation in a binocular field (MFBF), anti-suppression, and Gabor patches principles to enhance functional visual skills in the amblyopic eye without the need for patching [**66**]. These software solutions incorporate binocular and monocular activities that rewire the brain to improve vision in both eyes simultaneously, leading to greater compliance and faster results than patching. Conventional sports vision training includes manual drills such as saccadic and pursuit eye movements, reaction-time exercises, dynamic visual acuity training, eye-hand coordination exercises, GO-NO-GO therapies, and visual information-processing skills. These methods require extensive coaching and time, which can be challenging to implement across many athletes [**67**].

The use of advanced sports vision software, such as Vizual Edge, RightEye, Sports Vision, and Sports Vision Performance, has transformed athlete training by focusing on both holistic visual skills and sport-specific abilities, including reaction time, hand-eye coordination, and depth perception. These tools allow for individualized training tailored to the visual demands of different sports [**68**]. Computer vision and AI techniques also play a significant role in sports video analysis, aiding in tracking player movements, predicting ball trajectories, and analyzing team strategies. The Primary Sports Vision Performance Profile (PSVPP) remains an effective method for assessing and enhancing sports vision, underscoring the link between vision training and athletic performance [**69**]. Pencil push-ups and prism exercises are traditional methods for treating binocular vision disorders, particularly convergence insufficiency. These methods require daily practice and can be tedious for patients, often leading to low compliance [**70**]. SVI (Sanet Vision Integrator) and Vision Therapy System (VTS) use eye-tracking technology to improve eye coordination, offering precise assessments and individualized treatment protocols with real-time feedback. Studies show that software-based therapies yield faster results and better patient engagement than traditional techniques [**71**]. Visual-perceptual learning disorders are traditionally treated with visual-motor integration tasks, figure-ground discrimination exercises, and manual training to improve spatial awareness. While effective, these methods can be slow and require constant repetition. Software such as the Perceptual Therapy System (PTS) and CPT (computerized perceptual therapy system) provides interactive, tailored exercises designed to enhance visual information-processing skills [**72**]. These programs offer a faster and more engaging alternative to traditional perceptual learning methods, with a strong emphasis on neuroplasticity and visual processing. Post-surgical vision therapy, particularly after refractive surgery or ocular trauma, typically involves traditional exercises to restore visual acuity, eye alignment, and accommodation [**73**]. These include exercises such as lens flippers for accommodative recovery and saccadic charts for eye-movement training. Software like Eyebab and Neuro Visual Trainer provide post-surgical patients with exercises tailored to restore ocular function by stimulating neuroplasticity. Their eye-tracking technology also provides precise measurements of post-surgical visual performance and prescribes targeted exercises to improve eye movements and coordination [**74**]. Refractive errors, such as myopia and hyperopia, are commonly managed with corrective lenses or refractive surgeries. Traditional vision therapy exercises, such as near-far accommodation exercises, have also been used to reduce dependence on glasses, but the efficacy remains debatable. Software such as VisionUp and Vision Builder aims to improve visual function by enhancing accommodation and reducing refractive errors through perceptual learning. These systems use gamified tasks that promote visual flexibility, with some studies reporting improvements in accommodative function and a reduction in myopic progression [**75**].

### Clinical applications and efficacy

### Amblyopia treatment

Amblyopia, commonly known as “lazy eye”, is characterized by decreased vision in one eye due to abnormal visual development. Traditional treatments include patching or atropine penalization of the stronger eye, but vision therapy software offers an engaging alternative [**76**].

#### Clinical applications

**Dichoptic Training:** Software-based therapies often use dichoptic presentation, in which each eye is shown different images or stimuli. This forces the brain to use input from the weaker eye, thereby strengthening its neural connections [**77**]. **Game-Like Environments:** Interactive, game-based exercises help maintain patient engagement—especially important in pediatric populations—while reinforcing the use of the amblyopic eye [**78**]. **Improved Visual Acuity:** Studies have shown that consistent use of dichoptic training can lead to measurable improvements in visual acuity in amblyopic patients [**79**]. **Enhanced Binocular Integration:** Beyond acuity, these exercises can improve eye coordination, promoting better binocular vision and depth perception [**80**].

### Strabismus and binocular vision disorders

Strabismus involves misalignment of the eyes, leading to difficulties with binocular vision and often resulting in double vision or suppression of images from one eye. Vision therapy software is used to train proper eye alignment and improve visual integration [**81**].

#### Clinical applications

**Alignment and Fusion Exercises:** Many programs include exercises designed to help align the eyes and encourage the fusion of images from each eye, which is essential for proper depth perception [**82**]. **Feedback and Monitoring:** Real-time feedback from the software enables therapists to monitor patient progress and adjust exercise difficulty accordingly [**83**]. **Enhanced Eye Coordination:** Regular use has been linked to improved control over eye movements, reducing the severity of strabismus [**84**]. **Long-Term Visual Benefits:** Improved binocular coordination can lead to lasting changes in how visual information is processed, thereby improving overall visual performance [**85**].

### Convergence insufficiency and accommodative disorders

Convergence insufficiency is a condition in which the eyes have difficulty working together when focusing on a nearby object. At the same time, accommodative disorders involve problems with adjusting focus between near and distant distances. Vision therapy software targets these issues through specific exercises [**86**].

#### Clinical applications

**Near-Far Focus Exercises:** Programs include tasks that require shifting focus between objects at varying distances, strengthening the accommodative system [**87**]. **Convergence Drills:** Exercises designed to stimulate convergence help train the eyes to work together more effectively during near tasks, such as reading [**88**]. **Reduction of Visual Symptoms:** Patients often experience reduced symptoms such as eye strain, headaches, and blurred vision during near work [**6**]. **Improved Task Performance:** Enhanced convergence and accommodation lead to better performance in daily activities that require near vision, contributing to overall quality of life [**89**].

### Traumatic brain injury and post-concussion syndrome

Individuals who have sustained a traumatic brain injury or are experiencing post-concussion syndrome frequently encounter visual disturbances. These may include difficulties with eye tracking, focusing, and processing visual information [**90**].

#### Clinical applications

**Visual Processing Rehabilitation:** Vision therapy software can help retrain the brain to process visual information more efficiently through targeted exercises [**91**]. **Eye Movement Control:** Programs designed to improve saccadic and pursuit movements help restore smooth, coordinated eye movements, which are often impaired following TBI [**92**]. **Enhanced Recovery:** Early intervention with vision therapy software can accelerate and enhance visual function recovery, contributing to improved cognitive and motor performance [**93**]. **Symptom Alleviation:** Many patients report reduced symptoms such as blurred vision, dizziness, and headaches, which can significantly enhance their ability to resume daily activities [**94**].

### Myopia control and digital eye strain

Myopia and digital eye strain are increasingly prevalent, particularly with the rise in digital device use. Vision therapy software offers strategies to manage these conditions through exercises and environmental modifications [**95**].

#### Clinical applications

**Eye Exercises for Myopia Control:** Some vision therapy programs incorporate exercises aimed at reducing eye strain and, potentially, slowing myopia progression by improving focus and reducing accommodative stress [**96**]. **Digital Eye Strain Mitigation:** Programs can offer structured breaks, eye exercises, and visual activities to alleviate symptoms associated with prolonged screen time [**6**]. **Reduced Visual Fatigue:** Regular use of these exercises can help decrease symptoms of digital eye strain, including dryness, irritation, and fatigue [**97**]. **Potential Myopia Benefits:** While more research is needed, early evidence suggests that specific visual training programs may help slow myopia progression, especially when combined with other myopia management strategies [**98**].

Vision therapy software leverages the principles of neuroplasticity, adaptive training, and personalization to address a broad spectrum of visual disorders. Its clinical applications range from treating amblyopia and strabismus to aiding recovery after brain injuries and mitigating the effects of digital eye strain [**99**]. While individual responses to therapy may vary, the growing body of evidence supports the efficacy of these digital interventions in enhancing visual function, reducing symptoms, and ultimately improving patients’ quality of life. As technology and research continue to advance, vision therapy software is poised to become an even more integral part of vision rehabilitation strategies [**100**].

### Challenges of vision therapy software

Despite its advancements and accessibility, vision therapy software presents several challenges and limitations that must be addressed for optimal clinical integration [**101**].

### Effectiveness compared to traditional in-office therapy

While digital vision therapy offers an interactive and engaging platform, it may not fully replace in-office therapy sessions, where direct supervision and real-time adjustments by eye care professionals are crucial [**102**]. Certain complex cases, such as severe strabismus or neurovisual rehabilitation, may require in-person therapeutic interventions that cannot be replicated entirely through software-based programs. Clinical studies comparing digital vision therapy with traditional approaches are ongoing, and the long-term efficacy remains to be further validated [**103**].

### Need for supervision & professional guidance

Although many vision therapy programs offer self-guided exercises, proper execution and adherence to prescribed protocols require periodic supervision by an optometrist or vision therapist. Patients may struggle to identify and correct improper visual techniques without professional feedback, potentially limiting the effectiveness of therapy. Misuse or overuse of specific exercises without proper guidance may lead to visual discomfort, fatigue, or ineffective outcomes [**21**].

### Variability in patient response

The success of vision therapy software depends on patient-specific factors, including age, severity of the visual condition, cognitive engagement, and compliance with therapy. Some patients may show rapid improvement, while others may require extended therapy durations, necessitating continuous monitoring and individualized adjustments. Motivation and adherence to home therapy regimens can vary widely, affecting the consistency of results [**104**].

### Data privacy & ethical concerns

Vision therapy software often collects sensitive patient data, including visual performance metrics and health records, raising concerns about data security and privacy [**105**]. Compliance with regulations such as **the Health Insurance Portability and Accountability Act (HIPAA) {USA} and the General Data Protection Regulation** (GDPR) {Europe} is critical to protecting patient information and preventing data breaches. Ethical considerations arise regarding AI-driven therapy personalization—biases in algorithm training datasets could influence treatment recommendations, potentially leading to disparities in patient care [**106**]. While vision therapy software provides an innovative and accessible approach to vision rehabilitation, it is essential to address its challenges, including the need for professional supervision, variability in patient response, and data security concerns. A hybrid model that integrates digital tools with traditional in-office therapy may offer the best outcomes, ensuring both efficacy and safety in visual rehabilitation [**107**].

### Limitations of vision therapy software

Despite the advancements and benefits of vision therapy software, there are several limitations to consider. Not all patients may have access to the required technology, such as VR headsets or high-performance computers, particularly in under-resourced areas [**108**]. The technology may have a high learning curve. Some patients, particularly older adults, may struggle with the technology, which could reduce the effectiveness of the therapy. While software can track progress and offer real-time feedback, it cannot entirely replace the nuanced guidance and adjustments provided by an experienced therapist [**109**]. The cost of purchasing and maintaining advanced software and hardware can be prohibitive for smaller clinics or individual practitioners, limiting widespread adoption. Therapy delivered exclusively through software may lack the emotional support and motivation that in-person interactions with a therapist can provide [**110**].

### Recent research and evidence-based validation

Recent research has examined the efficacy of vision therapy software (VTS) across various visual disorders, providing insights from meta-analyses, clinical trials, comparative studies, and case reports [**111**].

#### Meta-analyses and clinical trials on VTS efficacy

A significant randomized clinical trial evaluated treatments for convergence insufficiency (CI) in children aged 9 to 17. Participants were assigned to one of four interventions: home-based pencil push-ups, home-based computer-based vergence/accommodative therapy with pencil push-ups, office-based vergence/accommodative therapy with home reinforcement, or office-based placebo therapy. The study aimed to determine the most effective treatment modality for symptomatic CI in children [**112**]. A meta-analysis assessed the efficacy of binocular treatments compared to traditional patching for amblyopia. The findings indicated no convincing evidence supporting binocular treatment as a complete replacement for conventional patching. However, binocular therapy may serve as a complementary approach in specific cases [**113**].

#### Comparative studies with traditional methods

A study compared virtual-reality-based vision therapy with traditional office-based vergence/accommodative therapy in patients with convergence insufficiency. Results demonstrated that virtual reality therapy significantly improved binocular vision functions and reduced symptoms, suggesting it as a viable alternative to conventional methods [**13**]. Research comparing home-based vision therapy, office-based vision therapy, and augmented office-based orthoptic treatment found that augmented office-based therapy was relatively more effective in adults with convergence insufficiency [**60**].

#### Case studies and patient outcomes

A case study evaluated the effectiveness of the CureSee vision therapy software in treating amblyopia. The study reported improvements in visual acuity and binocular functions, highlighting the potential of software-based interventions in amblyopia management [**114**]. These studies underscore the potential of vision therapy software as both a complementary and alternative approach to traditional methods. While some findings are promising, further research is essential to establish standardized protocols and determine long-term efficacy across diverse patient populations [**115**]. **Table 3** depicts the technological advancements in Vision Therapy from the 1980s to the 2050s.

**Table 3:** The technological advancements in Vision Therapy from the 1980s to the 2050s

S. No	Decade	Key Developments	Conditions Treated	Technology Used	Challenges
**1**	1980s	Traditional patching, eye exercises	Amblyopia, strabismus	Patching, manual exercises	Low patient compliance, limited tech
**2**	1990s	Introduction of basic computerized therapies	Amblyopia, convergence insufficiency	Basic computer programs	High cost, low access
**3**	2000s	Gamification, initial AI-based solutions	Amblyopia, CI, visual processing disorders	Gamified programs, AI-assisted software	Lack of long-term studies
**4**	2010s	VR-based therapy, AI advancements	Strabismus, amblyopia, sports vision	VR, advanced AI	High development costs, limited reach
**5**	2020s	Mobile-based apps, personalized treatment	Sports vision, neurological conditions	Mobile apps, AI, VR	Accessibility in rural areas
**6**	2030s-2050s	Fully personalized vision therapy	Full range of visual disorders	AI-driven platforms, VR, and AR integration	Scalability, ensuring long-term compliance

#### Future directions and innovations in Vision Therapy Software

With rapid advancements in technology, vision therapy software (VTS) is evolving to incorporate artificial intelligence (AI), virtual/augmented reality (VR/AR), wearable technology, and telemedicine. These innovations aim to enhance the effectiveness, accessibility, and personalization of vision therapy, making it more engaging and data-driven [**116**].

**Artificial intelligence and machine learning in vision therapy:** Artificial intelligence (AI) and machine learning (ML) are revolutionizing vision therapy by providing personalized treatment plans, adaptive difficulty levels, and predictive analytics [**117**]. **AI-Driven Personalized Therapy:** AI algorithms analyze patient progress, reaction times, and success rates to adjust therapy exercises dynamically. Machine learning can predict which treatments will be most effective based on historical patient data and refine therapy protocols accordingly [**118**]. **Automated Diagnosis and Progress Tracking:** AI-assisted vision screening tools can detect binocular vision disorders earlier by analyzing eye movement patterns and response times. AI-powered software can provide real-time progress analytics, helping doctors fine-tune therapy regimens remotely [**119**]. **Neural Adaptation Modeling:** Advanced neural network algorithms are being developed to simulate how the brain adapts to visual training, leading to more targeted exercises that enhance neuroplasticity. AI-powered platforms like RevitalVision use machine learning to optimize therapy based on patient-specific responses [**120**].

### Integration with Virtual Reality (VR) & Augmented Reality (AR)

Virtual Reality **(**VR) and Augmented Reality (AR) are transforming vision therapy by creating immersive, interactive, and controlled visual environments that enhance engagement and effectiveness [**121**].

**Virtual Reality for Amblyopia & Binocular Vision Disorders:** VR provides stereoscopic stimulation, which can train depth perception, vergence, and binocular fusion more effectively than traditional patching for amblyopia. VR-based programs like Vivid Vision use engaging games to encourage both eyes to work together, improving binocular vision [**122**]. **Augmented Reality (AR) for Real-World Applications:** AR overlays interactive visual exercises onto the patient’s real-world environment, making therapy more functional and practical. AR-based tools can help patients with visual tracking and saccadic dysfunctions by integrating digital visual targets into real-world tasks. Vivid Vision and Luminopia are pioneering VR therapy for amblyopia and binocular vision disorders [**123**].

### Wearable tech & eye-tracking for real-time feedback

Wearable devices and eye-tracking technology provide real-time monitoring of eye movements, pupil responses, and fixation accuracy, offering instant feedback and objective progress tracking [**124**]. **Smart Glasses for Vision Therapy:** Wearable devices like Ocutech Bioptics and eSight provide assistive vision therapy, enabling real-time vergence training and accommodation exercises [**125**]. **Eye-Tracking for Precision Therapy:** Eye-tracking technology integrated into headsets and smart screens helps monitor fixation stability, pursuit movements, and convergence insufficiency. Real-time feedback enhances patient engagement and therapist monitoring, ensuring exercises are performed correctly [**126**]. **Biofeedback & Neurostimulation:** Some wearables integrate neurofeedback to improve visual processing and cognitive engagement, optimizing therapy results. RightEye uses eye-tracking to assess and train visual skills, improving sports vision and rehabilitation outcomes [**127**].

### Telemedicine and remote monitoring advancements

The rise of telemedicine and remote vision therapy has expanded access to treatment, particularly for patients in underserved areas [**128**].

**Remote Vision Therapy Programs:** Online platforms allow patients to complete vision therapy sessions from home, with therapists monitoring their progress remotely. AI-based algorithms automatically adjust exercises based on user performance [**117**]. **Cloud-Based Data Sharing & Remote Assessments:** Cloud-based software enables real-time data sharing between patients and eye care professionals. Remote monitoring tools help optometrists and ophthalmologists track adherence and adjust treatment plans accordingly [**129**]. **Integration with Mobile Apps & Digital Health Records:** Mobile apps provide push notifications and gamified exercises to improve patient compliance and motivation. Digital integration with electronic health records (EHRs) allows seamless tracking of progress over time. AmblyoPlay and Optics Trainer offer telehealth-based vision therapy, allowing at-home training with remote monitoring [**130**]. The future of vision therapy software lies in AI-driven personalization, immersive VR/AR environments, wearable eye-tracking tools, and advances in telemedicine. These innovations are transforming how vision therapy is delivered, making it more accessible, engaging, and effective. As research continues, integrating these technologies with traditional in-office therapy will likely lead to better patient outcomes and expanded treatment options for various visual disorders [**131**].

### Pros and cons of Vision Therapy Software

The pros of vision therapy software include enhanced patient engagement, remote accessibility, cost-effectiveness, and objective progress tracking. Vision therapy software enables home-based therapy, reducing the need for frequent clinic visits. Increases access for patients in remote or underserved areas [**132**]. AI-driven platforms adjust difficulty levels based on patient performance. Provides tailored treatment plans for different visual conditions. Gamification and interactive elements enhance patient motivation, especially for children. Real-time feedback helps reinforce correct visual behavior. Allows clinicians to monitor progress remotely, improving adherence. Provides quantifiable data on improvement, helping in therapy adjustments [**117**]. VR/AR and eye-tracking enhance depth perception and dynamic training. Wearable devices improve real-time feedback and engagement. The initial device costs and potential lack of human interaction can be considered as drawbacks [**133**]. Despite these challenges, the long-term advantages position software-based approaches as the future of vision care. It may not fully replace in-office therapy, particularly for complex cases like severe strabismus. Patient response varies, requiring professional oversight for best results. Self-administered therapy risks incorrect execution without professional guidance. Patients, especially children, may lose interest or not adhere consistently [**134**]. Requires access to computers, VR headsets, or the internet, which may not be available to all patients. Some users (elderly, non-tech-savvy individuals) may struggle with digital interfaces. Software collects sensitive health data, requiring strict privacy regulations (HIPAA, GDPR). AI-driven recommendations may introduce bias in therapy adjustments. No universal clinical protocols exist for VTS implementation. More large-scale, long-term studies are needed to validate its efficacy [**108**].

Vision therapy software offers personalized, accessible, and engaging treatment options, enhancing patient compliance and expanding vision therapy beyond clinical settings. However, professional supervision, technological accessibility, and regulatory standardization remain key challenges. A hybrid approach, combining VTS with traditional in-office treatment, ensures the best outcomes for patients [**101**].

### Economic impact and patient compliance

The economic impact of vision therapy software is substantial, as the need for frequent in-office visits decreases, travel costs are reduced, and therapy can be conducted from home. Increased patient compliance is observed due to the engaging and accessible nature of software-based vision therapy [**135**]. Vision therapy software reduces the financial burden by minimizing the need for frequent in-office visits. Home-based therapy lowers travel costs and time commitments, making it more accessible. Subscription-based models and one-time payments offer greater flexibility in affordability for patients [**136**]. The demand for AI-driven and VR-based vision therapy is attracting increased investment in digital health. Telemedicine integration allows providers to expand services without significant infrastructure costs. Limited insurance reimbursement for digital treatment remains an important barrier. The initial cost of VR headsets, eye-tracking tools, and software subscriptions may limit adoption in specific populations [**137**]. Interactive exercises, reward systems, and AI-driven adaptations boost patient motivation. This is especially beneficial in pediatric and neuro-rehabilitation cases where engagement is crucial. VTS allows patients to perform therapy at their convenience, improving long-term adherence. Self-paced exercises with remote clinician monitoring enhance consistency and efficiency [**53**]. Lack of direct supervision can lead to inconsistent participation or improper technique. Patients may lose motivation over time without structured in-office follow-ups. Digital literacy and access issues could hinder compliance in elderly or lower-income populations [**138**].

## Conclusion

Vision therapy software is transforming vision rehabilitation by integrating AI, VR/AR, wearable technology, and telemedicine, making therapy more accessible, engaging, and data-driven. Research supports its efficacy in treating binocular vision disorders, amblyopia, and convergence insufficiency, though further long-term studies and standardization are needed. For optimal outcomes, a hybrid approach combining VTS with traditional in-office therapy under professional supervision is recommended. As technology advances, VTS will continue to redefine vision care, offering personalized, scalable, and effective solutions for patients worldwide. The hybrid approach, combining conventional exercises with these advanced digital solutions, may prove to be the most effective treatment model for a range of visual conditions, from amblyopia and strabismus to perceptual learning and post-surgical rehabilitation.
